# The Role of Multiparametric Magnetic Resonance in Volumetric Modulated Arc Radiation Therapy Planning for Prostate Cancer Recurrence After Radical Prostatectomy: A Pilot Study

**DOI:** 10.3389/fonc.2020.603994

**Published:** 2021-01-08

**Authors:** Angela Sardaro, Barbara Turi, Lilia Bardoscia, Cristina Ferrari, Giuseppe Rubini, Angela Calabrese, Federica Ammirati, Antonietta Grillo, Annamaria Leo, Filomenamila Lorusso, Antonio Santorsola, Antonio Amato Stabile Ianora, Arnaldo Scardapane

**Affiliations:** ^1^ Interdisciplinary Department of Medicine, Section of Radiology and Radiation Oncology, University of Bari “Aldo Moro”, Bari, Italy; ^2^ Radiation Oncology Unit, Azienda Ospedaliero-Universitaria Policlinico, Bari, Italy; ^3^ Radiation Therapy Unit, Department of Oncology and Advanced Technology, Azienda USL-IRCCS di Reggio Emilia, Reggio Emilia, Italy; ^4^ Nuclear Medicine Unit, Interdisciplinary Department of Medicine, University of Bari Aldo Moro, Bari, Italy; ^5^ Department of Radiology, IRCCS Istituto Tumori “Giovanni Paolo II”, Bari, Italy; ^6^ Department of Radiology, “Di Venere” Hospital of Bari, Bari, Italy

**Keywords:** prostate cancer recurrence, multiparametric magnetic resonance, radiotherapy, CT simulation, treatment planning system, dose–volume parameters, imaging registration

## Abstract

**Background and Purpose:**

Volumetric modulated arc radiotherapy (RT) has become pivotal in the treatment of prostate cancer recurrence (RPC) to optimize dose distribution and minimize toxicity, thanks to the high-precision delineation of prostate bed contours and organs at risk (OARs) under multiparametric magnetic resonance (mpMRI) guidance. We aimed to assess the role of pre-treatment mpMRI in ensuring target volume coverage and normal tissue sparing.

**Material and Methods:**

Patients with post-prostatectomy RPC eligible for salvage RT were prospectively recruited to this pilot study. Image registration between planning CT scan and T2w pre-treatment mpMRI was performed. Two sets of volumes were outlined, and DWI images/ADC maps were used to facilitate precise gross tumor volume (GTV) delineation on morphological MRI scans. Two rival plans (mpMRI-based or not) were drawn up.

**Results:**

Ten patients with evidence of RPC after prostatectomy were eligible. Preliminary data showed lower mpMRI-based clinical target volumes than CT-based RT planning (*p* = 0.0003): median volume difference 17.5 cm^3^. There were no differences in the boost volume coverage nor the dose delivered to the femoral heads and penile bulb, but median rectal and bladder V_70Gy_ was 4% less (*p* = 0.005 and *p* = 0.210, respectively) for mpMRI-based segmentation.

**Conclusions:**

mpMRI provides high-precision target delineation and improves the accuracy of RT planning for post-prostatectomy RPC, ensures better volume coverage with better OARs sparing and allows non-homogeneous dose distribution, with an aggressive dose escalation to the GTV. Randomized phase III trials and wider datasets are needed to fully assess the role of mpMRI in optimizing therapeutic strategies.

## Introduction

Prostate cancer (PC) is the second most frequent tumor diagnosis in men, accounting for 1,276,106 new cases reported worldwide in 2018, with a higher prevalence in developed countries (1, 2).

In Italy, PC has been estimated to account for 9.6% of all tumors diagnosed in the whole population and 18.5% of those in males in the last year (3).

Several factors may affect the risk of developing prostate cancer, such as age, black race, given the reported higher levels of androgens, dihydrotestosterone (DHT) and 5-alpha reductase than in Caucasian men (4); hormonal and genetic factors; a family history of PC (on both the paternal and maternal side); metabolic syndrome (although there is insufficient evidence to justify recommending lifestyle changes or a modified diet to lower this risk), and smoking (5).

In recent years, screening and early detection of PC by prostate-specific antigen (PSA) blood test has become one of the most controversial topics in the Uro-Oncology community due to increasing evidence of some cases of overdiagnosis. Despite this, PSA remains a better predictor of cancer than either clinical rectal examination or transrectal ultrasound; therefore, an individualised risk-adapted strategy for early detection is recommended (6).

PSA is also crucial in the follow-up after prostatectomy. Six weeks after primary surgery, PSA is expected to drop to undetectable values; consequently, PSA levels higher than 0.2 ng/ml in at least two subsequent samples are conventionally taken to define the condition of post-prostatectomy biochemical recurrence (BCR) of PC (7, 8).

Radiation therapy (RT) has become pivotal in the treatment of PC. It may represent a radical, exclusive approach for organ-confined or locally advanced disease, and may also be performed as adjuvant or salvage treatment following radical prostatectomy, in cases with adverse pathological features (pT3a-pT3b-pT4 staging high- and very high-risk PC, positive surgical margins), biochemical failure and/or macroscopic evidence of disease recurrence (8, 9).

In the field of External Beam Radiotherapy (EBRT), advances in rotational, intensity-modulated delivery techniques with volumetric modulated arc irradiation (VMAT) have made it possible to individualize the radiation dose distribution to the prostate volume while sparing the surrounding normal tissues and organs, thus optimizing treatment efficacy and minimizing genitourinary (GU) and gastrointestinal (GI) toxicity (10, 11).

Magnetic resonance imaging (MRI) has gained increasing interest for the pre-treatment assessment of prostate cancer. Advances in diagnostic procedures, improving diagnostic reliability for primary and recurrent PC (RPC), have allowed a more accurate detection of prostatic lesions (6). In particular, given the better soft tissue contrast provided by anatomic MRI, the accuracy of prostate tumor identification, anatomic location and characterization in terms of extraprostatic extension and seminal vesicle involvement now ranges from 69 to 90% (12, 13). The addition of functional sequences, such as diffusion-weighted imaging (DWI), dynamic contrast-enhanced (DCE) imaging and MR spectroscopy has further improved the performance of MRI imaging in tumor detection (12, 13).

In this scenario, multiparametric MR imaging (mpMRI) has emerged as helpful in the precise identification of tumor site and extent, extraprostatic and/or seminal vesicles involvement. mpMRI is also reported to allow the precise location of a recurrent prostate tumor, and so to assess if the disease relapse is strictly limited to the prostate bed since it appears as a T2-weighted (T2w) isointense to hyperintense lesion close to the surgical scar, with rapid early enhancement and washout on DCE MR sequences (14). Such a highly accurate delineation of the prostate contours under mpMRI guidance has also given rise to radiation treatment optimization: so-called dose-painting, that is targeting prostate tumor sites with a higher radiation dose, with or without a dose gradient on the lower-risk prostate/prostate bed areas, while guaranteeing maximum sparing of the bladder and rectum (13, 15). Most of the available literature is focused on the identification of dominant, intraprostatic lesions to define dose-escalation protocols in the radical setting (16, 17). On the contrary, data on the identification of post-prostatectomy RPC lesions to be safely boosted for an ablative eradication treatment protocol are still lacking. We carried out a prospective pilot study with the aim of assessing the role of pre-treatment mpMRI in target volume delineation and treatment planning for recurrent prostate cancer after radical prostatectomy, in terms of target volume coverage and normal tissue sparing.

## Materials and Methods

### Patient Selection and Treatment

Patients with post-prostatectomy BCR as per Phoenix criteria (7), and with macroscopic evidence of RPC, presenting at our Institution and eligible for salvage EBRT, were prospectively recruited to the study. A staging workup with 11C-Choline PET-CT was performed in cases with two consecutive PSA values ≥1 ng/ml, to exclude nodal and/or systemic metastatization.

Radiation treatment was performed using Image-guided radiotherapy (IGRT), VMAT technique, with daily cone beam CT scans for monitoring.

All the recruited patients underwent pelvic mpMRI before EBRT planning, regardless of any other morphological and/or functional workup already performed for diagnostic purposes. Then, planning pelvic CT scan with 3 mm slice was acquired, both for mpMRI and CT, in conventional, supine position.

The present study was carried out in accordance with the principles of Good Clinical Practice, conforming to the ICH GCP guidelines and the ethical principles contained in the Helsinki declaration.

Since the recruitment period started during the rapid COVID-19 spread in Italy, all the diagnostic and treatment procedures described below were performed in accordance with the Italian Government official recommendation statements and Italian Association of Radiotherapy and Clinical Oncology (AIRO) tips for the management of oncological patients in the context of the COVID-19 pandemic (18, 19).

### mpMRI Protocol and Image Interpretation

The mpMRI was performed with a 1.5 T scanner (Philips Achieva 1.5), using a 16-channel surface coil in supine position according to the PIRADS 2.1 protocol.

The inhibition of intestinal peristalsis was guaranteed through the intramuscular injection of 10 ml/mg of N-butyl scopolamine (Buscopan, Boehringer Ingelheim, Germany), before the MRI test.

The imaging protocol consisted of the following sequences:

- T2-weighted Turbo Spin Echo (TSE), on the axial, coronal and sagittal planes.
- Axial FOV (AP 160, RL 160 mm, FH 82 mm), matrix 212 × 206 × 25 slices, NSA (number of signals) three;
- Coronal FOV (AP 66 mm, RL 160 mm, FH 160), matrix 200 × 195 × 20 slices, NSA (number of signals) two;
- Sagittal FOV (AP 160, RL 82 mm, FH 160 mm), matrix 200 × 199 × 25 slices, NSA (number of signals) two;
- TE 110 ms, TR shortest, section thickness 3 mm, 24–30 sections, acquisition time 3–3.5 min.
- T2-weighted Turbo Spin Echo (TSE), on the axial planes with wide view to evaluate lymph node involvement, FOV (AP 300, RL 300 mm, FH 258 mm), matrix 332 × 299 × 40 slices, NSA (number of signals) one;
- Dynamic THRIVE SPAIR, on the axial plane, FOV (FH 75mm, RL 200, AP 200 mm), matrix 112 × 171 × 25 slices, NSE (number of signals) one; TE/TR shortest, thickness 6 mm, 20 acquisition (10 s for each acquisition).
- DWI, on the axial plane, TE/TR shortest, section thickness 3 mm, with B value of 0.700, 1000 and 1400, FOV (RL 160, AP 160mm, F H 99 mm), matrix 64 × 56 × 25 slices, NSE (number of signals) one; ADC maps were subsequently calculated.

All the images were reviewed and interpreted by two radiologists (with 20 and 4 years’ experience, respectively).

The RPC diagnosis was based on the presence of solid nodules on T2w scan, with close evaluation of the most frequent location on the vesicourethral anastomosis, discretely vascularized after the injection of contrast agent, showing signal restriction in the DWI sequences and a low signal on the ADC (apparent diffusion coefficient) map.

The presence of any other pelvic tumor mass and enlarged hypogastric, obturator, iliac and sacral lymph nodes (diameter > 5 mm) was checked.

Areas of signal intensity restriction on DWI/ADC images, as well as enhancing areas with no visible pathologic tissue on morphologic T2w images, were also recorded.

### Planning Computed Tomography Acquisition Protocol

Before CT simulation, all patients underwent rectal emptying (using an enema 2–3 h before the procedure) and comfortable bladder filling (complete urination 30 min before CT scan, then drinking 500 ml of water until CT execution), in order to ensure inter-fraction setup reproducibility during treatment delivery and improved sparing of organs at risk (OARs).

CT simulation was acquired with patients in supine position, hands over the chest, using foot lock and kneefix support systems. Longitudinal alignment along the sternum and navel and transverse alignment at the level of the pubic symphysis were ensured, while the field depth was defined at the patient’s hemi-thickness. Skin reference points were marked at the laser crossings, with corresponding placement of three radiopaque markers.

A control scan was first performed to assess the correct alignment of the patient on the CT table, and the longitudinal alignment was checked on the anterior topogram. Once the patient’s position had been verified, CT scan with 3 mm slices was acquired. At the end of the procedure, the correct position of the radiopaque markers on the zero slice, adequate bladder filling, and rectal emptying were checked.

### Radiation Treatment Planning

Target volume delineation and RT planning were performed using the Monaco^®^ HD Treatment Planning System (TPS) 5,11,03 by Elekta. In cases of MR evidence of RPC lesions, image registration between the planning CT scan and T2w plus DWI mpMRI images was performed. Two sets of volumes were contoured, with and without mpMRI-guidance. Gross tumor volume (GTV) was outlined in both the CT simulation and the T2w MR scan, DWI images and related apparent diffusion coefficient (ADC) maps were used to facilitate the precise GTV contouring onto the morphological MR scans. Clinical target volume (CTV) was defined as the prostate fossa, and contoured as per the Kirsty et al. consensus definition for the anatomic boundaries of the prostate bed (20): the superior boundary was the superior surgical clips (if present) or 5 mm above the inferior border of the vas deferens; the inferior boundary was 8 mm below the vesicourethral anastomosis or the top of the penile bulb; the posterior 15 mm of the bladder wall was taken as the anterior, cranial boundary; the posterior edge of the pubis symphysis up to the top of the pubis symphysis as the anterior, caudal boundary; the lateral, cranial boundary was the sacrorectogenitopubic fascia, lateral to the neurovascular structures; the posterior, cranial boundary was the mesorectal fascia; the lateral, caudal boundary was the medial border of the levator ani and obturator internus muscles; the anterior border of the rectal wall and levator ani muscle was the posterior, caudal boundary; a 10-mm extension of the outlined boundaries was made beyond the GTV and the visible surgical clips located outside the boundaries, if present, except for high lymphadenectomy vessel clips. An isotropic, 5 mm expansion was applied to the CTV to obtain the Planning treatment volume (PTV) (prostate bed), and to the GTV to obtain the PTV (boost). OARs (bladder, rectum, penile bulb, femoral heads) were delineated in both mpMRI and planning CT scan.

Two rival plans were drawn up, one per each set of volumes, taking into account the dose constraints for limiting normal tissue toxicity based on the quantitative Analysis of Normal Tissue Effects in the Clinic (QUANTEC) (21) and the Radiation Therapy Oncology Group (RTOG) GU consensus (22). The GU OAR dose constraints applied for VMAT inverse treatment planning are summarized in **Table 1**. Dose prescription, dose recording and reporting were performed as per ICRU Report 83 (23). We ensured that the same target volumes coverage was obtained in the rival plans.

**Table 1:** QUANTEC dose constraints for inverse treatment planning (21, 22).

Organs at Risk	Volume segmented	Dose (Gy) or dose/volume parameters
**Rectum**	Whole organ	V_50Gy_ <50%
		V_65Gy_ <25%
		V_70Gy_ <20%
**Bladder**	Whole organ	V_55Gy_ <50%
		V_65Gy_ <50%
		V_70Gy_ <35%
**Penile bulb**	Whole organ	Mean dose to 95% of gland <50 Gy
		D_60-70%_ <70 Gy
**Femoral heads**	Whole organ	V_50Gy_ <5%

The prescribed dose was 70 Gy in 35 daily fractions on the prostate lodge, with a sequential boost or higher doses targeting any macroscopic evidence of RPC at the pre-treatment mpMRI.

### Statistical Analysis

A sample size of at least 10 RPC patients was arbitrarily defined, since this was a pilot, prospective trial and no similar study designs with which to compare accrual evaluation have been reported in literature. Statistical analysis is intended as descriptive for future findings and data integration. For the same purpose, the size of the obtained CTV (prostate bed) (cm^3^), the dose covering 98% of the PTV (boost) (D_98%_), the volume (%) of rectum receiving 50, 65, and 70 Gy (V_50Gy_, V_65Gy_ and V_70Gy_), the volume (%) of bladder receiving 55, 65 and 70 Gy (V_55Gy_, V_65Gy_ and V_70Gy_), the volume (%) of femoral heads receiving 50 Gy (V_50Gy_), the mean dose to 95%, and the dose delivered to 70% of the penile bulb volume (D_70%_) were chosen as referral parameters for statistical comparison between the rival mpMRI and non-mpMRI plans.

Statistical analysis was performed using SPSS statistical software v25.0. To describe the data, median and ranges were used and proportions and percentages for categorical variables. Differences between two-sample central tendencies were assessed with two independent samples t-test. A *p* value of <0.05 was considered statistically significant.

## Results

From April 2020 to October2020, a total of 17 patients fitted the selection criteria.

Choline-PET restaging was negative in all the patients who performed it.

Negative pre-treatment mpMRI confirmed biochemical recurrence in 7 (41.2%) patients.

Ten (58.8%) patients showed macroscopic disease recurrence at the pre-EBRT mpMRI, PSA values ranging from 0.52 to 6.9 ng/ml. Among these, all but one underwent a total dose of 70 Gy—2-Gy/fraction on the prostate fossa, then a sequential boost on the RPC lesion(s) was delivered according to the following schedules: additional 2 Gy per five fractions (80 Gy in total) for 3 patients (30%), 6 Gy in three fractions (total 76 Gy) for two (20%) patients, and 2 Gy per four fractions (total 78 Gy) for four (40%) of them; one (10%) patient underwent a total dose of 80 Gy on the entire prostate bed.

**Figure 1:**
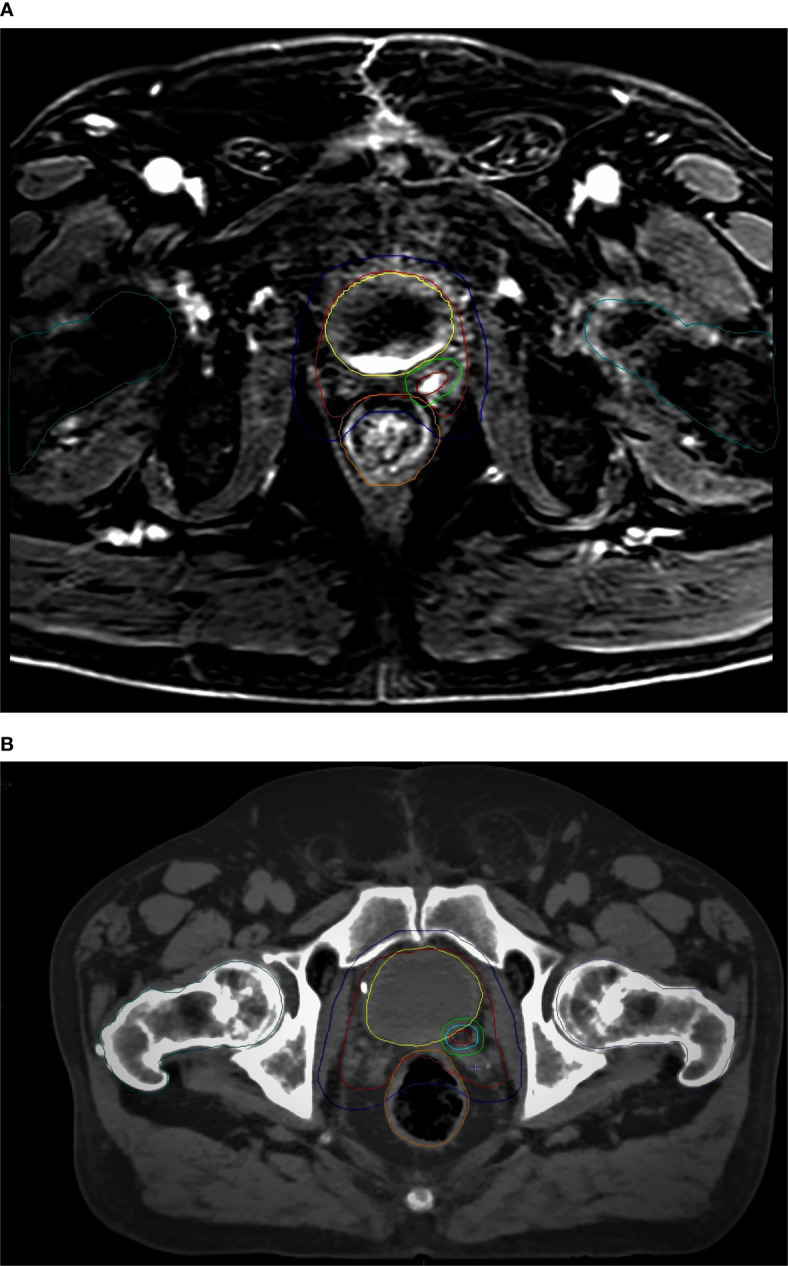
mpMRI-based **(A)** *vs* CT-based **(B)** CTV/PTV (prostate bed) and OARs delineation: red, CTV (prostate bed); blue, PTV (prostate bed); yellow, bladder; orange, rectum; dark green, femoral heads.

**Figure 2:**
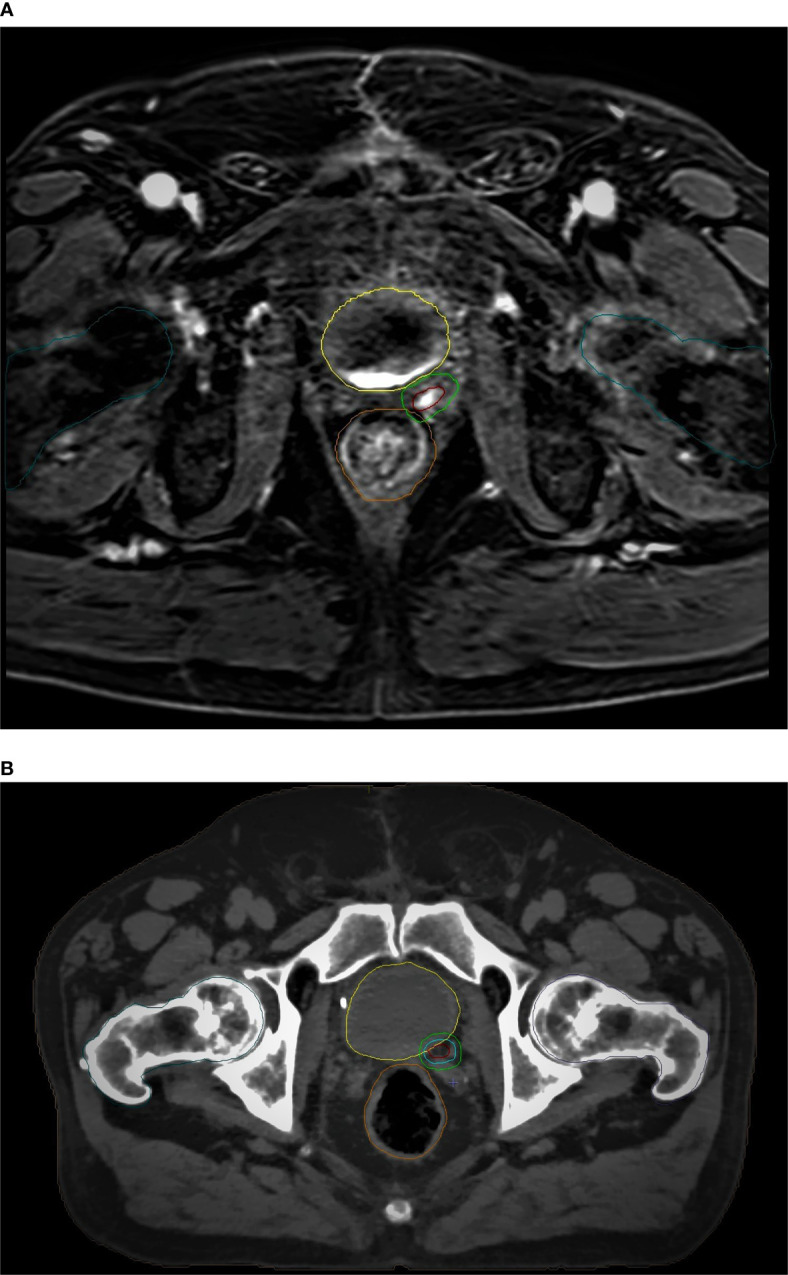
mpMRI-based **(A)** *vs* CT-based **(B)** GTV/PTV (boost) and OARs delineation: brown, GTV; light green, PTV (boost); yellow, bladder; orange, rectum; dark green, femoral heads.

An example of CT-based target volume and OAR delineation with and without mpMRI co-registration in our series is reported in **Figure 1** and **Figure 2**.

Preliminary dosimetric comparison between mpMRI-based and non-mpMRI-based treatment plans was made in the 10 enrolled patients with macroscopic RPC. The results obtained are reported in **Table 2** and **Table 3**. Median delineated CTV (prostate bed) was 15.7 cm^3^ in size (range 5.6–30.7) on the T2w MRI scan, compared to median 33.4 cm^3^ (range 19.5–51.0) for those outlined on the CT simulation only, with a median difference of 17.5 cm^3^ (range 8.3–26.7) between the two contour sets (*p* = 0.0003). No substantial differences were found in terms of the boost volume coverage, median D_98%_ for PTV (boost) was 76.3 Gy (range 74–78.5) for mpMRI-based delineation and 76.1 Gy (range 73.8–77.2) for CT-based delineation only (*p* = 0.376).

**Table 2:** Dosimetric comparison between mpMRI-based and non-mpMRI-based RT plans: target volume coverage (significant correlations shown in bold).

	CT-based RT plan	mpMRI-based RT plan	Difference	P value
**CTV (prostate bed) (cm^3^)**				
Patient 1	19.50	11.20	8.30	
Patient 2	34.70	8.00	26.70	
Patient 3	51.00	30.72	20.28	
Patient 4	24.30	5.64	18.66	
Patient 5	32.22	21.08	11.14	**0.0002**
Patient 6	32.35	15.20	17.15	
Patient 7	34.40	16.30	18.10	
Patient 8	30.30	12.40	17.90	
Patient 9	43.60	27.00	16.60	
Patient 10	35.70	19.30	16.40	
**PTV (boost) D_98%_ (Gy)**				
Patient 1	74.10	74.90	0.80	
Patient 2	74.00	74.90	0.90	
Patient 3	75.70	76.00	0.30	
Patient 4	73.80	74.00	0.20	
Patient 5	76.36	78.5	2.14	0.376
Patient 6	76.10	76.20	0.10	
Patient 7	76.10	76.40	0.30	
Patient 8	77.20	77.30	0.10	
Patient 9	76.90	77.20	0.30	
Patient 10	77.00	78.10	1.10	

**Table 3:** Dosimetric comparison between mpMRI-based and non-mpMRI-based RT plans: high-doses OAR sparing (significant correlations shown in bold; outrange values highlighted).

	CT-based RT plan	mpMRI-based RT plan	Difference	*P* value
**Rectum V_70Gy_ (%)**				
Patient 1	5.10	4.90	0.20	
Patient 2	14.50	9.80	4.70	
Patient 3	16.70	13.80	2.90	
Patient 4	13.40	7.70	5.70	
Patient 5	13.00	9.50	3.50	**0.005**
Patient 6	13.30	8.10	5.20	
Patient 7	14.40	9.00	5.40	
Patient 8	12.60	8.70	3.90	
Patient 9	15.30	11.12	4.18	
Patient 10	13.60	10.30	3.30	
**Bladder V_70Gy_ (%)**				
Patient 1	**37.30**	**35.00**	2.30	
Patient 2	14.50	10.30	4.20	
Patient 3	33.30	33.00	0.30	
Patient 4	24.90	22.90	2.00	
Patient 5	28.00	22.70	5.30	0.210
Patient 6	25.20	21.30	3.90	
Patient 7	27.20	22.50	4.70	
Patient 8	24.20	20.40	3.80	
Patient 9	28.20	23.30	4.90	
Patient 10	28.20	22.40	5.80	

As to OARs, no differences were found in terms of femoral heads and penile bulb sparing, nor differences in rectum V_50Gy_ and V_65Gy,_ bladder V_55Gy_ and V_65Gy_, respectively (data not shown). However, we recorded a median rectal V_70Gy_ 4% (range 0.2–5.7) smaller for treatment delineation on T2w MRI scans, with a median V_70Gy_ of 9.2% (range 4.9–13.8) for mpMRI-based RT plans, and of 13.5% (range 5.1–16.7) for non-mpMRI based RT plans (*p* = 0.005). Likewise, there was a median bladder V_70Gy_ of 22.6% (range 10.3–35) for mpMRI-based RT plans, compared to median 27.6% (range 14.5–37.3) for CT-based RT plans, with a median bladder V_70Gy_ difference of 4% (range 0.3–5.8) between the two rival plans (*p* = 0.210).

## Discussion

The addition of image registration with pre-treatment mpMRI to CT-based target volume delineation has dramatically improved the accuracy of treatment planning for prostate cancer. MR imaging is well known to provide better soft tissue contrast than conventional morphological imaging techniques such as ultrasound or computed tomography (CT). For this reason, mpMRI may ensure a proper definition of the pelvic organs anatomy, of the prostate and prostate bed boundaries, so that several publications have highlighted a smaller prostate volume delineated on MRIs than on CTs (13–17). Moreover, the addition of functional sequences like DWI, DCE, and MR spectroscopy further improved the precise location and characterization of primary and recurrent prostate tumor lesions, even in the post-prostatectomy setting (14). Such findings, together with the possibility of dose-painting and advances in stereotactic EBRT and VMAT delivery techniques, have opened out a new era allowing highly non-homogeneous dose distributions, with aggressive dose escalation to the GTV while contemporarily reducing the dose to lower-risk areas of the prostate, and improving OARs sparing (13, 15).

Post-prostatectomy recurrent tumors may be found at the urethrovesical anastomosis, seminal vesicles bed, bladder posterior wall or rectal anterior wall. Boonsirikamchai et al. retrospectively showed >90% accuracy of DCE MR scans in the detection of prostate bed recurrences (14). Currently, mpMRI is the only imaging technique recommended by the European Society of Urogenital Radiology (ESUR) for the detection of pelvic post-prostatectomy RPC in patients with PSA rising to conventional biochemical relapse values (0.2–2 ng/ml) (24, 25), given its higher sensitivity in detecting local lesions, especially small ones (<10 mm) with low blood PSA levels (26). Detection rates of 84–95% have been reported using endorectal coil-mpMRI for PSA values >1 ng/ml, while other retrospective studies and a metanalysis reported recurrence rates ranging from 24 to 91%. This variability is due to the retrospective nature of the study designs and extremely heterogeneous sample sizes and procedures (15).

The addition of functional DWI and DCE perfusion scans to the morphological T2w imaging dramatically improved the performance of MRI in detecting malignant lesions, with their typical, albeit relatively variable, early uptake and early washout of the contrast material (15).

DWI imaging is also powerful in detecting tumor masses since they appear as highly cellular tissues. However, in the prostate setting, the ADC alone, derived for absolute quantification, may be vague and difficult to interpret. mpMRI can overcome the problem of the unavoidable heterogeneity of the ADC maps due to the well-known highly heterogeneous cell density between malignant and benign prostate areas, which may obscure small lesions or part of tumor masses (27).

The precise location and segmentation of a tumor mass have given rise to better patient selection for focal therapy (28). In the field of EBRT, Stoyanova and colleagues analyzed mpMRI of 65 planned and treated prostate cancer patients and found they could detect the tumor burden by including ADC and DCE-MRI information, imported in a DICOM-RT ready format, into the radiotherapy TPS (29). They subsequently created an automated, mpMRI-based, quantitative method to guide dose-escalation to more aggressive prostate tumor masses, referenced to the prostatectomy Gleason score (30). This means that mpMRI-guided, precise delineation of tumor boundaries may allow the complete eradication of disease through highly-escalated dose delivery, leading to radiation doses of up to 80 Gy on the high-risk GTVs while ensuring lower doses to the low-risk, less aggressive prostate areas, with better OARs dose saving and hence limited treatment-related toxicity. In this regard, the ongoing prospective, phase II trials, hypo-FLAME Trial and DELINEATE Trial, both reached their primary endpoint in terms of acceptable acute toxicity with simultaneous focal boosting to the mpMRI-detected macroscopic tumor(s) in addition to whole gland prostate irradiation (31, 32). Thus, in the setting of salvage RT for macroscopic, local recurrence of PC after radical prostatectomy, such findings are likely to improve the efficacy and safety of radiotherapy: firstly, thanks to a dose-escalated boost over the mpMRI-delineated RPC lesions, in addition to the 64 to 70 Gy-standard irradiation of the prostate fossa (33, 34); secondly, thanks to the precise segmentation of prostate bed recurrent masses with the help of mpMRI co-registration, enabling stereotactic salvage treatments to be performed in the attempt to provide a better local control than the conventional, normofractionated RT protocols (35).

On the other hand, there are some intrinsic limitations due to the non-linear information content of the registered MR images compared to planning CT scan, as the former is usually performed regardless of the radiation treatment position, with a different appearance of the surrounding hollow OARs, often with the use of an endorectal coil which is helpful in the correct delimitation of prostate boundaries from the rectal wall, but in contrast with the EBRT patients setup requiring an empty rectum and comfortable bladder filling (36). In this regard, Couñago and colleagues first found a significantly higher probability of radiological evidence of local recurrence at the pre-treatment mpMRI without endorectal coil in patients undergoing salvage EBRT for biochemical relapse after prostatectomy with PSA doubling time >14 months (adjusted Odds ratio (OR) 7.12, p = 0.01) and/or PSA levels >0.5 ng/ml (adjusted OR 6.25, p = 0.02) (37). Ciardo et al. also described a multimodal, voxel-based and contrast enhancement-based deformable registration procedure, with acceptable accuracy despite varying setups in MRI (17).

Despite the limitation of the few analyzed cases and intrinsic biases due to the pilot study design, our results are in line with the scarce available literature, and support the use of pre-treatment mpMRI to better assess the prostate tumor phenotype and personalize radiation treatment segmentation and dose prescription. In our series, mpMRI was acquired without an endorectal coil, in view of the patient treatment position. The possibility of EBRT treatment planning with the help of image registration with pre-treatment mpMRI allowed high-precision matching and target volume delineation. This made it possible to deliver doses up to 80 Gy to the RPC lesions or to the whole prostate bed without compromising treatment safety and tolerability, possibly improving the sparing of the surrounding normal tissues and OARs.

Another limitation of our study is the unavoidable, intrinsic variability of the CTVs and GTVs due to the extension of the RPC lesions and patients anatomy, affecting the outlined volumes. However, the mpMRI-based CTV (prostate bed) were significantly smaller than the CT-based ones in all the recruited patients, with a significant median reduction of 17.5 cm^3^. These findings support the more accurate identification and delineation of the volumes of interest through the better soft tissue contrast provided by mpMRI, together with the possible better dose saving to the surrounding normal tissues.

Equally importantly, dosimetric evaluation showed that rectum and bladder sparing were most evident in the analysis of high doses. Since the macroscopic RPC is the volume of interest, receiving the higher radiation dose up to 76–80 Gy, the greatest difference in terms of normal tissues sparing is reasonably expected at the highest dose levels. Our results seem to confirm this trend.

International guidelines recommend rectum V_70Gy <_20% and bladder V_70Gy <_35% (21, 22). These dose constraints were respected in both rival plans for all the recruited patients, except for one case of bladder V_70Gy_ 37.3% in the non-mpMRI-based RT plan, which we could reduce to below threshold (V_70Gy_ 35%) with the help of mpMRI registration (**Table 3**). Our series revealed a median 4% reduction in rectum and bladder V_70Gy_, although the latter was not statistically significant. Such differences may be relevant to prevent exceeding or not the recommended dose constraints, and this may have a non-negligible impact on treatment tolerance and toxicity. Therefore, if such results should be confirmed in a wider series, it may be reasonable to define the percentage of OAR volume receiving more than a 70 Gy dose as the most useful parameter to ensure dose saving to the rectum and bladder during post-prostatectomy prostate bed irradiation.

Further studies are warranted to fully assess the role of mpMRI in optimizing therapeutic strategies, especially in the field of post-prostatectomy recurrent PC, as well as the ability of combined anatomic and functional MR scans to provide biological and pathophysiological information on prostate cancer behavior, and so radiation resistance (38).

The completion of our statistical analysis on a wider dataset will strengthen the statistical power of our series and possibly provide a definitive validation of our promising findings.

Noteworthily, in the era of next-generation IGRT, where MR-Linac EBRT has been gaining increasing interest in the field of prostate cancer (39–41), radiation treatment planning with mpMRI appears as a cost-effective procedure and may contribute to ensure quality oncological healthcare in the South of Italy.

In conclusion, mpMRI may improve the accuracy of radiation treatment planning in the setting of salvage EBRT for recurrent prostate cancer after prostatectomy, provide high precision target volumes and OAR delineation, thus ensuring better target volume coverage with better OAR sparing. The addition of mpMRI to conventional, CT-based EBRT planning may also improve the optimization of dose distribution, as it allows a dose reduction to lower-risk areas of the prostate fossa, together with an aggressive dose escalation to the macroscopic RPC lesions, while reducing the dose delivered to the surrounding normal tissues.

In the future perspective of patient-tailored medicine, prospective, randomized, phase III studies, and validation of our findings on a wider dataset are needed to complete the assessment of the power of T2w/ADC-based target volume segmentation and dose prescription in improving the accuracy and precision, hence safety and effectiveness, of radiation treatment delivery for primary and recurrent prostate cancer.

## Data Availability Statement

The datasets presented in this article are not readily available because the present prospective study is currently ongoing. Requests to access the datasets should be directed to Angela Sardaro, angela.sardaro@uniba.it.

## Author Contributions

AnS developed the project, analyzed the data, and edited the manuscript. BT edited the manuscript. LB reviewed the literature and wrote the manuscript. CF edited the manuscript. GR analyzed the data, performed accuracy check, and edited the manuscript. AC wrote the manuscript. FA collected the data. AG collected the data and edited the manuscript. AL collected and managed the data. FL edited the manuscript. AS analyzed the data. AI interpreted the data and edited the manuscript. ArS developed the project, checked the data integrity, checked the data analysis accuracy, and edited the manuscript. All authors contributed to the article and approved the submitted version.

## Conflict of Interest

The authors declare that the research was conducted in the absence of any commercial or financial relationships that could be construed as a potential conflict of interest.

## References

[1] Bray F Ferlay J Soerjomataram I Siegel RL Torre LA Jemal A Global cancer statistics 2018: GLOBOCAN estimates of incidence and mortality worldwide for 36 cancers in 185 countries. CA Cancer J Clin (2018) 68(6):394–424. 10.3322/caac.21492 30207593

[2] Arnold M Karim-Kos HE Coebergh JW Byrnes G Antilla A Ferlay J Recent trends in incidence of five common cancers in 26 European countries since 1988: Analysis of the European Cancer Observatory. Eur J Cancer (2015) 51(9):1164–87. 10.1016/j.ejca.2013.09.002 24120180

[3] AIOM - AIRTUM SIAPEC-IAP I Numeri del Cancro in Italia 2020. Intermedia EDITORE (2020). Available at: https://www.aiom.it/wp-content/uploads/2020/10/2020_Numeri_Cancro-operatori_web.pdf.

[4] Dess RT Hartman HE Mahal BA Soni PD Jackson WC Cooperberg MR Association of Black Race With Prostate Cancer-Specific and Other-Cause Mortality. JAMA Oncol (2019) 5(7):975–83. 10.1001/jamaoncol.2019.0826 PMC6547116 31120534

[5] Merriel SWD Funston G Hamilton W Prostate Cancer in Primary Care. Adv Ther (2018) 35(9):1285–94. 10.1007/s12325-018-0766-1 PMC6133140 30097885

[6] Mottet N Bellmunt J Bolla M Briers E Cumberbatch MG De Santis M EAU-ESTRO-SIOG Guidelines on Prostate Cancer. Part 1: Screening, Diagnosis, and Local Treatment with Curative Intent. Eur Urol (2017) 71(4):618–29. 10.1016/j.eururo.2016.08.003 27568654

[7] Amling CL Bergstralh EJ Blute ML Slezak JM Zincke H Defining prostate specific antigen progression after radical prostatectomy: what is the most appropriate cut point? J Urol (2001) 165(4):1146–51. 11257657

[8] Cornford P van den Bergh RCN Briers E Van den Broeck T Cumberbatch MG De Santis M EAU-EANM-ESTRO-ESUR-SIOG Guidelines on Prostate Cancer. Part II—2020 Update: Treatment of Relapsing and Metastatic Prostate Cancer. Eur Urol (2020). 10.1016/j.eururo.2020.09.046 33039206

[9] Pisansky TM Thompson IM Valicenti RK D’Amico AV Selvarajah S Adjuvant and Salvage Radiotherapy after Prostatectomy: ASTRO/AUA Guideline Amendment 2018-2019. J Urol (2019) 202(3):533–8. 10.1097/JU.000000000000029510 PMC8680266 31042111

[10] Kuban DA Tucker SL Dong L Starkschall G Huang EH Cheung MR Long-term results of the M. D. Anderson randomized dose-escalation trial for prostate cancer. Int J Radiat Oncol Biol Phys (2008) 70(1):67–74. 10.1016/j.ijrobp.2007.06.054 17765406

[11] Peeters ST Heemsbergen WD Koper PC van Putten WL Slot A Dielwart MF Dose-response in radiotherapy for localized prostate cancer: results of the Dutch multicenter randomized phase III trial comparing 68 Gy of radiotherapy with 78 Gy. J Clin Oncol (2006) 24(13):1990–6. 10.1200/JCO.2005.05.2530 16648499

[12] Delongchamps NB Rouanne M Flam T Beuvon F Liberatore M Zerbib M Multiparametric magnetic resonance imaging for the detection and localization of prostate cancer: combination of T2-weighted, dynamic contrast-enhanced and diffusion-weighted imaging. BJU Int (2011) 107(9):1411–8. 10.1111/j.1464-410X.2010.09808.x 21044250

[13] Boonsirikamchai P Choi S Frank SJ Ma J Elsayes KM Kaur H MR imaging of prostate cancer in radiation oncology: what radiologists need to know. Radiographics (2013) 33(3):741–61. 10.1148/rg.333125041 23674772

[14] Boonsirikamchai P Kaur H Kuban DA Jackson E Hou P Choi H Use of maximum slope images generated from dynamic contrast-enhanced MRI to detect locally recurrent prostate carcinoma after prostatectomy: a practical approach. AJR Am J Roentgenol (2012) 198(3):W228–36. 10.2214/AJR.10.6387 22358019

[15] Counago F Sancho G Catala V Hernandez D Recio M Montemuino S Magnetic resonance imaging for prostate cancer before radical and salvage radiotherapy: What radiation oncologists need to know. World J Clin Oncol (2017) 8(4):305–19. 10.5306/wjco.v8.i4.305 PMC5554874 28848697

[16] Steenbergen P Haustermans K Lerut E Oyen R De Wever L Van den Bergh L Prostate tumor delineation using multiparametric magnetic resonance imaging: Inter-observer variability and pathology validation. Radiother Oncol (2015) 115(2):186–90. 10.1016/j.radonc.2015.04.012 25935742

[17] Ciardo D Jereczek-Fossa BA Petralia G Timon G Zerini D Cambria R Multimodal image registration for the identification of dominant intraprostatic lesion in high-precision radiotherapy treatments. Br J Radiol (2017) 90(1079):20170021. 10.1259/bjr.20170021 28830203 PMC5963367

[18] COVID-19, raccomandazioni per i pazienti oncologici. Available at: http://www.salute.gov.it/portale/nuovocoronavirus/dettaglioNotizieNuovoCoronavirus.jsp?lingua=italiano&menu=notizie&p=dalministero&id=4200 (Accessed April 1, 2020).

[19] Filippi AR Russi E Magrini SM Corvò R Letter from Italy: First practical indications for radiation therapy departments during COVID-19 outbreak. Int J Radiat Oncol Biol Phys (2020) 107(3):597–9. 10.1016/j.ijrobp.2020.03.007 PMC7141469 32199941

[20] Wiltshire KL Brock KK Haider MA Zwahlen D Kong V Chan E Anatomic boundaries of the clinical target volume (prostate bed) after radical prostatectomy. Int J Radiat Oncol Biol Phys (2007) 69(4):1090–9. 10.1016/j.ijrobp.2007.04.068 17967303

[21] Marks LB Yorke ED Jackson A Ten Haken RK Constine LS Eisbruch A Use of normal tissue complication probability models in the clinic. Int J Radiat Oncol Biol Phys (2010) 76(3 Suppl):S10–9. 10.1016/j.ijrobp.2009.07.1754 PMC4041542 20171502

[22] Lawton CAF Michalski J El-Naqa I Buyyounouski MK Lee WR Menard C RTOG GU Radiation oncology specialists reach consensus on pelvic lymph node volumes for high-risk prostate cancer. Int J Radiat Oncol Biol Phys (2009) 74(2):383–7. 10.1016/j.ijrobp.2008.08.002 PMC2905150 18947938

[23] ICRU ICRU report Vol. 83. Bethesda: International Commission on Radiation Units and Measurements. Prescribing, recording, and reporting photon-beam intensity-modulated radiation therapy (IMRT). J ICRU (2010) 10(1). 10.1093/jicru/ndq001

[24] Buyyounouski MK Hanlon AL Eisenberg DF Horwitz EM Feigenberg SJ Uzzo RG Defining biochemical failure after radiotherapy with and without androgen deprivation for prostate cancer. Int J Radiat Oncol Biol Phys (2005) 63(5):1455–62. 10.1016/j.ijrobp.2005.05.053 16169682

[25] Barentsz JO Richenberg J Clements R Choyke P Verma S Villeirs G ESUR prostate MR guidelines 2012. Eur Radiol (2012) 22(4):746–57. 10.1007/s00330-011-2377-y PMC3297750 22322308

[26] Panebianco V Sciarra A Lisi D Galati F Buonocore V Catalano C Prostate cancer: 1HMRS-DCEMR at 3T versus [(18)F]choline PET/CT in the detection of local prostate cancer recurrence in men with biochemical progression after radical retropubic prostatectomy (RRP). Eur J Radiol (2012) 81(4):700–8. 10.1016/j.ejrad.2011.01.095 21330082

[27] Borren A Moman MR Groenendaal G Boeken Kruger AE van Diest PJ van der Groep P Why prostate tumour delineation based on apparent diffusion coefficient is challenging: an exploration of the tissue microanatomy. Acta Oncol (2013) 52(8):1629–36. 10.3109/0284186X.2013.787164 23621751

[28] Wysock JS Lepor H Multi-parametric MRI imaging of the prostate-implications for focal therapy. Transl Androl Urol (2017) 6(3):453–63. 10.21037/tau.2017.04.29 PMC5503978 28725587

[29] Stoyanova R Sandler K Pollack A Delineation and visualization of prostate cancer in multiparametric MRI. Pract Radiat Oncol (2013) 3(2 Suppl 1):S30–1. 10.1016/j.prro.2013.01.105 24674544

[30] Stoyanova R Chinea F Kwon D Reis IM Tschudi Y Parra NA An Automated Multiparametric MRI Quantitative Imaging Prostate Habitat Risk Scoring System for Defining External Beam Radiation Therapy Boost Volumes. Int J Radiat Oncol Biol Phys (2018) 102(4):821–9. 10.1016/j.ijrobp.2018.06.003 PMC6245650 29908220

[31] Draulans C van der Heide UA Haustermans K Pos FJ van der Voort van Zyp J De Boer H Primary endpoint analysis of the multicentre phase II hypo-FLAME trial for intermediate and high risk prostate cancer. Radiother Oncol (2020) 147:92–8. 10.1016/j.radonc.2020.03.015 32247206

[32] Murray JR Tree AC Alexander EJ Sohaib A Hazell S Thomas K Standard and Hypofractionated Dose Escalation to Intraprostatic Tumor Nodules in Localized Prostate Cancer: Efficacy and Toxicity in the DELINEATE Trial. Int J Radiat Oncol Biol Phys (2020) 106(4):715–24. 10.1016/j.ijrobp.2019.11.402 31812718

[33] Shelan M Odermatt S Bojaxhiu B Nguyen DP Thalmann GN Aebersold DM Disease Control With Delayed Salvage Radiotherapy for Macroscopic Local Recurrence Following Radical Prostatectomy. Front Oncol (2019) 9:12. 10.3389/fonc.2019.00012 30873377 PMC6403145

[34] Dirix P van Walle L Deckers F Van Mieghem F Buelens G Meijnders P Proposal for magnetic resonance imaging-guided salvage radiotherapy for prostate cancer. Acta Oncol (2017) 56(1):27–32. 10.1080/0284186X.2016.1223342 27587084

[35] Francolini G Jereczek-Fossa BA Di Cataldo V Simontacchi G Marvaso G Zerella MA Stereotactic radiotherapy for prostate bed recurrence after prostatectomy, a multicentric series. BJU Int (2020) 125(3):417–25. 10.1111/bju.14924 31608534

[36] Hanvey S Sadozye AH McJury M Glegg M Foster J The influence of MRI scan position on image registration accuracy, target delineation and calculated dose in prostatic radiotherapy. Br J Radiol (2012) 85(1020):e1256–62. 10.1259/bjr/26802977 23175491 PMC3611732

[37] Counago F del Cerro E Recio M Diaz AA Marcos FJ Cerezo L Role of 3T multiparametric magnetic resonance imaging without endorectal coil in the detection of local recurrent prostate cancer after radical prostatectomy: the radiation oncology point of view. Scand J Urol (2015) 49(5):360–5. 10.3109/21681805.2015.1004643 25652562

[38] Zamboglou C Eiber M Fassbender TR Eder M Kirste S Bock M Multimodal imaging for radiation therapy planning in patients with primary prostate cancer. Phys Imaging Radiat Oncol (2018) 8:8–16. 10.1016/j.phro.2018.10.001 33458410 PMC7807571

[39] McPartlin AJ Li XA Kershaw LE Heide U Kerkmeijer L Lawton C MR-Linac consortium: MRI-guided prostate adaptive radiotherapy - A systematic review. Radiother Oncol (2016) 119(3):371–80. 10.1016/j.radonc.2016.04.014 27162159

[40] Mannerberg A Persson E Jonsson J Gustafsson CJ Gunnlaugsson A Olsson LE Dosimetric effects of adaptive prostate cancer radiotherapy in an MR-linac workflow. Radiat Oncol (2020) 15(1):168. 10.1186/s13014-020-01604-5 32650811 PMC7350593

[41] Dunlop A Mitchell A Tree A Barnes H Bower L Chick J Daily adaptive radiotherapy for patients with prostate cancer using a high field MR-linac: Initial clinical experiences and assessment of delivered doses compared to a C-arm linac. Clin TranslRadiat Oncol (2020) 23:35–42. 10.1016/j.ctro.2020.04.011 PMC7210377 32395640

